# Non‐native grazers affect physiological and demographic responses of greater sage‐grouse

**DOI:** 10.1002/ece3.9325

**Published:** 2022-09-20

**Authors:** Tessa Behnke, Phillip Street, Scott Davies, Jenny Q. Ouyang, James S. Sedinger

**Affiliations:** ^1^ Program in Ecology, Evolution, and Conservation Biology University of Nevada–Reno Reno Nevada USA; ^2^ Department of Natural Resources and Environmental Science University of Nevada–Reno Reno Nevada USA; ^3^ Department of Biological Sciences Quinnipiac University Hamden Connecticut USA

**Keywords:** avian physiology, *Centrocercus urophasianus*, corticosterone, drought, grazing, greater sage‐grouse, non‐native ungulate

## Abstract

Non‐native ungulate grazing has negatively impacted native species across the globe, leading to massive loss of biodiversity and ecosystem services. Despite their pervasiveness, interactions between non‐native grazers and native species are not fully understood. We often observe declines in demography or survival of these native species, but lack understanding about the mechanisms underlying these declines. Physiological stress represents one mechanism of (mal)adaptation, but data are sparse. We investigated glucocorticoid levels in a native avian herbivore exposed to different intensities of non‐native grazing in the cold desert Great Basin ecosystem, USA. We measured corticosterone, a glucocorticoid in feathers for a large sample (*n* = 280) of female greater sage‐grouse (*Centrocercus urophasianus*) from three study areas in Northern Nevada and Southern Oregon with different grazing regimes of livestock and feral horses. We found that greater feral horse density was associated with higher corticosterone levels, and this effect was exacerbated by drought conditions. Livestock grazing produced similar results; however, there was more model uncertainty about the livestock effect. Subsequent nesting success was lower with increased feather corticosterone, but corticosterone levels were not predictive of other vital rates. Our results indicate a physiological response by sage‐grouse to grazing pressure from non‐native grazers. We found substantial among‐individual variation in the strength of the response. These adverse effects were intensified during unfavorable weather events, highlighting the need to reevaluate management strategies in the face of climate change.

## Graphical abstract

Understanding the mechanisms behind the influence of non‐native species on Greater Sage‐grouse may help us mitigate population declines. We found that feral horses, domestic cattle and drought conditions increase physiological stress, as measured by feather corticosterone.

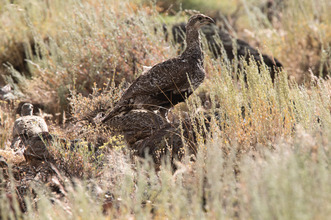

## 1 INTRODUCTION

Glucocorticoid hormones (GCs) link a vertebrate's internal condition and its external environment (Sapolsky et al., 2000). Baseline levels of GCs have been related to components of fitness in several species (Bonier et al., 2009; Cyr & Romero, 2007; Hau et al., 2010; Henderson et al., 2017; Madliger & Love, 2016; Sorenson et al., 2017; Wingfield & Sapolsky, 2003). The Cort‐Fitness Hypothesis, proposed by Bonier et al. (2009), suggests that elevated baseline levels of GCs reflect a physiological response to environmental or social resource limitation that in turn results in lower reproductive investment or survival, thereby lowering fitness. Alternatively, Bonier et al. (2009) also propose the Cort‐Adaptation Hypothesis, in which elevated GCs help direct resources toward reproduction. Existing data are conflicting as to the nature of this relationship (Injaian et al., 2020; Madliger & Love, 2016; Sorenson et al., 2017), leaving us with no consensus on the interplay between GCs and fitness. Understanding is poor in part due to lack of context (Romero, 2004; Vera et al., 2017) about the specific environment as experienced by wild individuals.

Invasive or introducted species serve as novel stressors that can detrimentally increase physiological stress in co‐occurring native species, impacting survival and/or reproduction (Graham et al., 2012; Narayan et al., 2015; Santicchia et al., 2018; Van Zwol et al., 2012). Introduced non‐native ungulates, both domestic and wild, are known to degrade habitats for native species across numerous ecosystems (Dettenmaier et al., 2017; Eldridge et al., 2020; Spear & Chown, 2009; Volery et al., 2021), thereby having the potential to chronically elevate GC levels. Grazing by domestic cattle (*Bos bos*) has been a prominent land use in the western United States for more than a century (Bureau of Land Management, 2020b). Feral horses (*Equus ferus caballus*) have increased dramatically over the past two decades, largely associated with sociopolitical resistance to removing horses from rangelands (Garrott, 2018). Both livestock and horses graze plants that are preferred by native herbivores because of their higher nutritional content (Hanley & Hanley, 1982; Scasta et al., 2016; Veblen et al., 2015). Livestock and horses also cause physical damage to riparian areas (Batchelor et al., 2015; Beever & Herrick, 2006; Beschta et al., 2014; Boyd et al., 2017; Dobkin et al., 1998), which provide essential foods and water during dry periods in the cold deserts of western North America (Batzer & Baldwin, 2012; Donnelly et al., 2018). Soil trappling and compaction can occur from overuse, negatively affecting ecosystem processes (Beever & Herrick, 2006; Byrnes et al., 2018). Such impacts are likely to be exacerbated when seasonal moisture is reduced because primary productivity is lower under such conditions in already dry environments (Chambers et al., 1999; Donnelly et al., 2018; Zeigenfuss et al., 2014).

**FIGURE 1:**
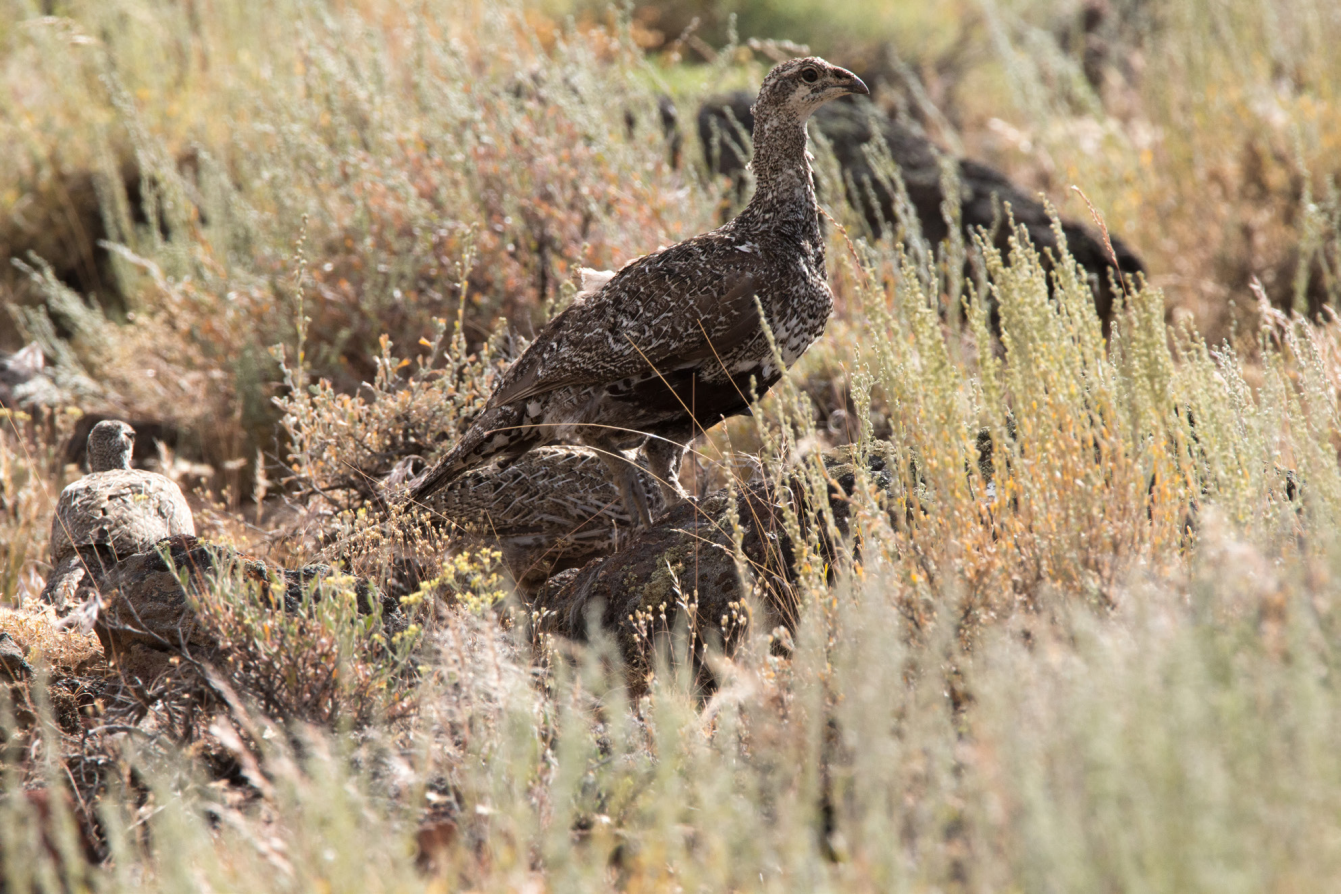
Photo of female greater sage‐grouse with brood in dry late summer habitat, northwestern Nevada.

Greater sage‐grouse (*Centrocercus urophasianus*; hereafter, “sage‐grouse”; Figure 1) are herbivorous birds that are sympatric with feral horses and/or livestock throughout their range in the western United States (Schroeder et al., 2020). They rely on sagebrush as their main food source outside the breeding season but expand their diet during breeding to include insects and other plants (Rowland et al., 2006). Sage‐grouse are highly selective of specific sagebrush plants because individual shrubs can vary in nutrient content and anti‐herbivore secondary compounds (Frye et al., 2013; Remington & Braun, 1985).

Following nesting in early spring, adult female sage‐grouse and their offspring feed on forbs and associated invertebrates (Casazza et al., 2011; Gregg et al., 2008; Gregg & Crawford, 2009). As the offspring age, the days get longer, temperature increases, precipitation decreases, and by early summer (June), these critical foods are available primarily in higher elevation sites or riparian areas. Such areas represent as little as 2% of the landscape (Atamian et al., 2010; Donnelly et al., 2016). Growth rate of young, which influences both prefledging and postfledging survival (Blomberg, Gibson, et al., 2014; Blomberg, Sedinger, et al., 2014; Gibson et al., 2016), is directly dependent on the quality and quantity of food (Blomberg, Poulson, et al., 2013; Smith et al., 2019). It seems likely that adult females also restore some depleted nutrient reserves during this period because forbs represent the highest protein content foods females encounter (Gregg et al., 2008), and protein is a limiting nutrient for herbivorous birds (Sedinger, 1997). These high‐quality foods potentially regulate individual fitness and population dynamics of sage‐grouse (Kane et al., 2017). Consequently, non‐native ungulates have the potential to negatively influence sage‐grouse through their influence on key food plants (e.g., sagebrush and forbaceous vegetation) and seasonal habitats used by sage‐grouse. In fact, recent analyses by Coates et al. (2021) demonstrate that abundance of feral horses is negatively correlated with the rate of growth of local sage‐grouse populations.

Assessment of the potential effects of domestic cattle and feral horses on sage‐grouse has primarily been through the lens of habitat modification or behavioral disturbance (Beck & Mitchell, 2000; Boyd et al., 2014; Dahlgren et al., 2015; Dettenmaier et al., 2017; Williamson et al., 2020). Direct assessment of the effects of livestock or horses on fitness, demography, and population dynamics of sage‐grouse is lacking. Possible physiological mechanisms for demographic effects resulting from coexistence with these non‐native ungulates are also largely unaddressed (Monroe et al., 2017).

We measured variation in corticosterone levels, the predominant GC in birds, in feathers of female sage‐grouse across a gradient of livestock, and feral horse densities in multiple years that varied in precipitation to assess the elevation of GC levels in relation to the abundance of livestock and feral horses under variable climatic conditions. Feather corticosterone (fCort) is an indicator of chronic stress, which may alter an individual's ability to allocate resources to reproduction or survival over time. In house sparrows (*Passer domesticus*) and Eurasian Sparrowhawks (*Accipiter nisus*), higher fCort levels in feathers were associated with lower survival in the following season (Koren et al., 2012; Monclus et al., 2017). High GC concentrations in feathers led to a higher likelihood of skipping breeding in the following breeding season for harlequin ducks (*Histrionicus histrionicus*) (Hansen et al., 2016) and successfully breeding giant petrels (*Macronectes* spp.) had 1.5 times higher fCort than non‐breeders (Crossin et al., 2013), indicating some cost to reproduction. Other studies have found no support for links between fCort and fitness (Henderson et al., 2017; Madliger & Love, 2016), which suggests that these relationships may be context‐dependent.

We assessed potential sources of chronic stress and the demographic consequences for female sage‐grouse by testing the relationships between fCort and important demographic rates, including adult survival, probability of initiating breeding, nest success, and probability of producing fledged young. We utilized ongoing management actions, e.g., feral horse removals, and grazing regimes as quasi‐experimental treatments across a large area of sage‐grouse habitat. Under the Cort‐Fitness Hypothesis, we predicted horse and cow densities would be related to increases in fCort, followed by declines in survival and breeding of sage‐grouse.

## 2 MATERIALS AND METHODS

### 2.1 Study areas

**FIGURE 2:**
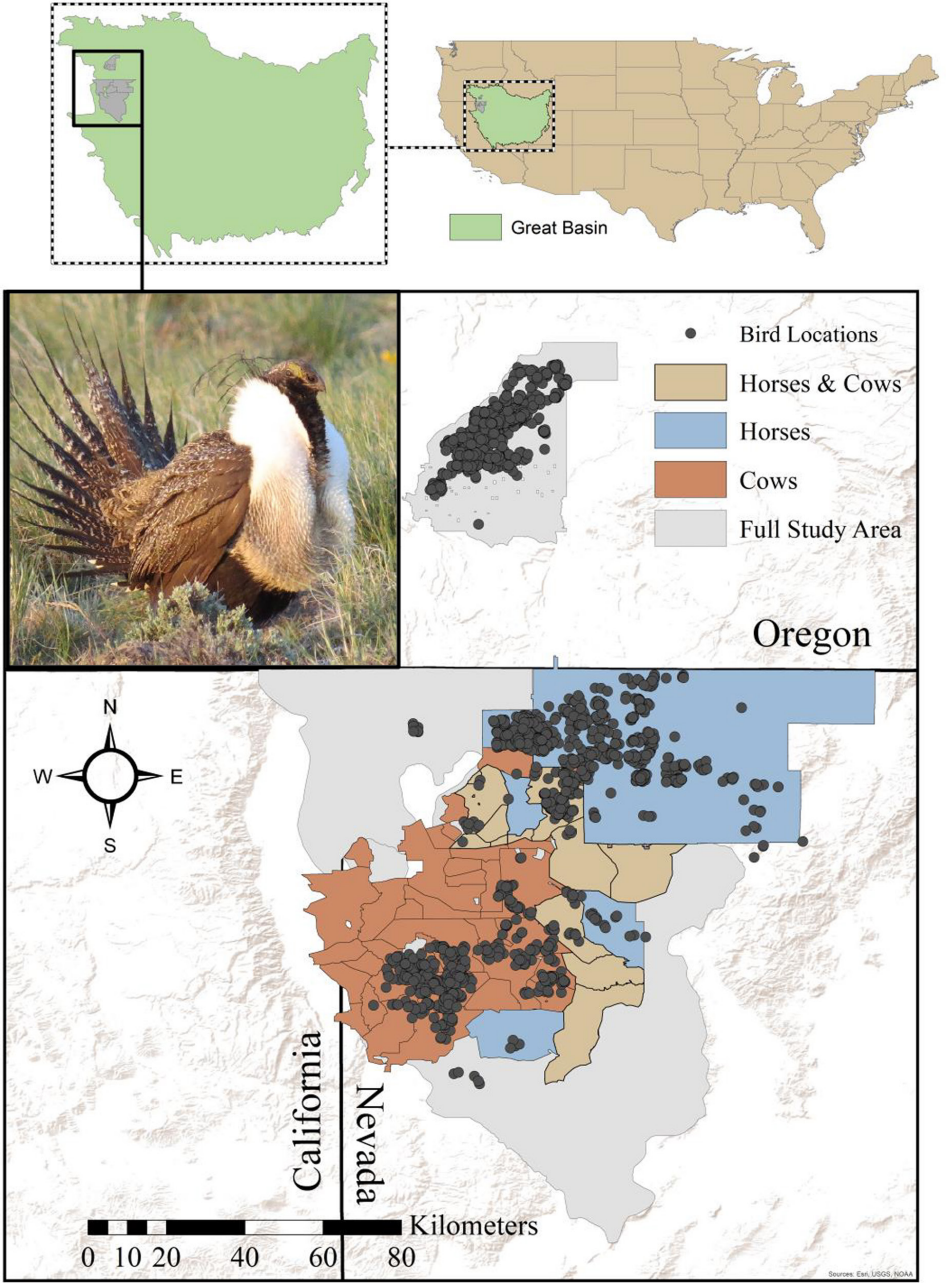
Study area map showing areas with horses, cows, or both during the course of the study. Bird locations include all known locations of the birds with fCort measurements. Hart Mountain was the northern‐most area, while Sheldon was the approximately rectnaangular area on the Oregon border. BLM managed lands containing cattle allotments and horse management areas were the remainder of the area. Hart Mountain did have a few horses (<31) in 2012–2013, but these were not in the same areas as monitored sage‐grouse.

We studied sage‐grouse in three study regions in the northern Great Basin, USA (Figure 2). Hart Mountain National Antelope Refuge (hereafter Hart Mountain) in southcentral Oregon encompasses over 112,500 hectares and ranges in elevation from 1360 to just over 2445 m at the top of Warner Peak (U.S. Fish and Wildlife Service, 2020a , 2020b). Domestic livestock grazing ceased on Hart Mountain in 1991, and feral horses occur only in very low numbers outside areas where we studied sage‐grouse. Sheldon National Wildlife Refuge (hereafter Sheldon) in northern Nevada includes over 231,840 hectares (U.S. Fish and Wildlife Service, 2020a, 2020b). The lowest elevation is approximately 1300 m at the bottom of the Thousand Creek Gorge, and the highest peak is Catnip Mountain, which reaches just over 2220 m. Domestic livestock were removed from Sheldon in 1994. Feral horses were abundant, but nearly all feral horses were removed in 2014–2015. The Bureau of Land Management (BLM)‐managed portion of our study is delineated by the Vya and Massacre Sage‐grouse Population Management Units (hereafter Vya‐Massacre), which encompass approximately 711,200 hectares in northwestern Nevada and northeastern California (Nevada Department of Wildlife, 2017). There are 22 grazing allotments for domestic cattle, as well as seven Herd Management Areas (HMAs) for feral horses, which overlap in the BLM portion of our study area (Figure 2).

### 2.2 Field methods

We trapped yearling (ca. 10 months old at the time of capture) and adult female sage‐grouse near active breeding leks (Gibson & Bradbury, 1987) during March of 2013–2017. We captured birds at night using spotlights (Giesen et al., 1982) and handheld nets. Once captured, we banded females with unique metal bands (National Band and Tag; size 14) and fit them with 22 g very high‐frequency necklace‐style radio‐collars (VHF; Advanced Telemetry Systems). We measured culmen using dial calipers (±1 mm), tarsus using dial calipers (±1 mm), flat wing using a board (±1 mm), and weighed each individual (±1 g). We aged birds as yearlings or adults based on plumage characteristics (Crunden, 1963). During capture, we plucked two breast feathers, which are thought to be grown in fall (Clait Braun, personal comm.) from each individual. We labeled feathers by individual and stored them frozen in small plastic storage bags (2013–2016) or dry in small paper coin envelopes (2017–2019). For this analysis, we used feathers collected from Hart Mountain (2013–2015), Sheldon (2013–2017), and Vya‐Massacre (2013–2017).

We monitored females every 2–3 days via ground telemetry during the early nesting period, to document nesting and success or failure of nests. For females that had successful nests, we began monitoring their broods within the first 2–3 days after chicks hatched (Street et al., 2022). We performed weekly checks of these broods, to count the surviving chicks up to 42–49 days of age. For the remaining months of the year, we employed aerial telemetry, approximately monthly, to assess live/dead status and location.

### 2.3 Additional data

We used reported data to assess horse and livestock usage, in this area, cows, across our study region (Figure 2). For cows, we used BLM data for each allotment, which included the number of cows present and the amount of time spent in the allotment (BLM Applegate Field Office unpublished data). To account for variation in the number of days, cows were actually on each allotment, we multiplied the number of cows by number of days they were on an allotment, then divided by the size of the area to produce a variable reflecting cow use days per unit area. For horses, we used annual population estimates reported for each Herd Management Area (HMA; Bureau of Land Management, 2020a). Because horses are on the landscape year‐round, we multiplied number of horses by 365 days and divided by the size of the HMA. Thus, our variables for analysis were horse and cow use days per square kilometer.

Our study region experienced significant variation in precipitation among years (150–410 mm). We used cumulative precipitation from October to September, referred to as the “northern water year,” as a measure of annual drought conditions (Huntington et al., 2017; PRISM Climate Group, 2011). Because invasive annual grasses displace native vegetation and can be detrimental to sage‐grouse fitness and population dynamics (Blomberg et al., 2012), we included invasive annual grass cover as an explanatory variable in some candidate models of fCort levels. We used year‐specific layers of estimated invasive annual grass percent cover created by Boyte et al., 2019, which in this area is primarily cheatgrass (*Bromus tectorum*), for this purpose.

Because feathers were collected at the time of capture, in late winter or early spring, the exact geographic locations of the birds during feather growth were unknown. Site fidelity of sage‐grouse for specific seasonal habitats is high (Fischer et al., 1993), thus we used locations collected after capture as an index of where the female was the prior season. We extracted values for horse and cow density, drought, and annual grass cover for each individual sage‐grouse at each location and averaged the covariate values across all locations. We used the locations for the year corresponding to feather growth, rather than the year the bird was captured for these estimates.

We calculated body condition as: residuals of body mass regressed on female structural size, a principal components score of wing and tarsus lengths (Blomberg, Gibson, et al., 2014; Freeman & Jackson, 1990). When measurements were missing (*n* = 16), we assigned the individual the mean condition value. As noted above, measurements were conducted at the time of capture so our analyses of condition assessed whether fCort was predictive of body condition a few months after feather growth.

### 2.4 Hormone assays

We extracted corticosterone from feathers following Bortolotti et al. (2008), with some modifications. We first removed the calamus before measuring the length of each feather. We cut each feather into pieces <5 mm^2^, mixed the pieces with 7 mL of HPLC grade methanol, and incubated the samples in a sonicating water bath at room temperature for 30 min. After sonication, we incubated the samples overnight in a shaking water bath at 50°C. To separate the feather pieces and methanol, we used vacuum filtration with grade 4 filter paper (Whatman). We also washed the sample tube, feather pieces, and filter apparatus twice with 2 mL of methanol, with washes added to the methanol extract. We dried the methanol extracts under air in a 50°C heat block, reconstituted the extracts in assay buffer provided with the assay kit (see below), and froze them until assay.

We quantified corticosterone using enzyme‐linked immunoassay according to the manufacturer's instructions (Enzo Life Sciences). To validate this assay, we verified that a serially diluted sample was parallel with the corticosterone standard curve. The average intra‐ and inter‐assay coefficients of variation were 4.7% and 16.9%, respectively. The average assay sensitivity was 8.3 pg/mL. Because hormone deposition occurs in a time‐dependent fashion during feather growth over several days to weeks (Bortolotti, Marchant, Blas, & Cabezas, 2009; Bortolotti, Marchant, Blas, & German, 2008), we normalized all final corticosterone values by feather length.

### 2.5 Corticosterone model

We analyzed fCort levels in a Bayesian framework using JAGS UI (Kellner, 2015; Plummer, 2003) in Program R (R Core Team, 2019). We modeled fCort rounded to the nearest pg/mm for each bird *i*, with negative binomial variation approximated by a mean fCort level (μ), and shape term (r) to account for the highly skewed distribution. For all models we considered r to be constant. We modeled individual variation among μ with covariates of age (yearling or adult) and body condition. We included horse and cow densities, precipitation, and annual grass cover to assess the potential for impacts of grazing by non‐native ungulates on fCort. We included interaction terms for horses × precipitation, cows × precipitation, and horses × annual grass to allow for spatial–temporal variation in the effects of non‐native grazers in response to variation in climate or ecological conditions. Continuous covariates were *z*‐standardized so that the estimates of each beta value are comparable to each other. Thus, our full model, for each individual female, i, was,

$$\begin{array}{cc} \log\left(\mu_{i}\right)= & \beta_{0}+\beta_{\mathrm{age}}\times {\mathrm{age}}_{i}+\beta_{\text{body condition}}\times {\text{body condition}}_{i}\\ & +\beta_{\mathrm{cow}}\times {\mathrm{cow}}_{i}+\beta_{\text{horse}}\times {\text{horse}}_{i}+\beta_{\text{precipitation}}\times {\text{precipitation}}_{i}\\ & +\beta_{\text{annual grass}}\times {\text{annual grass}}_{i}+\beta_{\text{horse},\text{precipitation}}\times {\text{horse}}_{i}\times {\text{precipitation}}_{i}\\ & +\beta_{\mathrm{cow},\text{precipitation}}\times {\mathrm{cow}}_{i}\times {\text{precipitation}}_{i}+\beta_{\text{horse},\text{annual grass}}\times {\text{horse}}_{i}\times {\text{annual grass}}_{i} \end{array}$$

**FIGURE A1:**
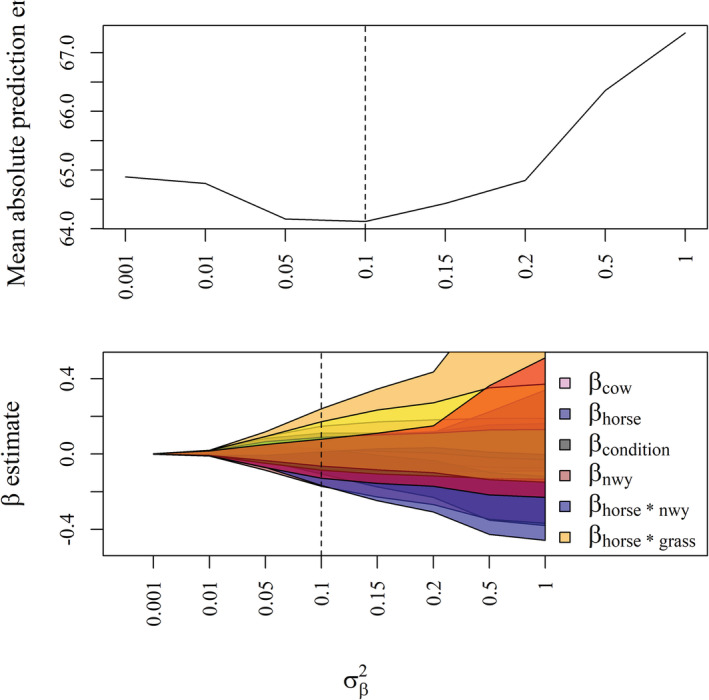
Sigma optimization. Upper plot shows the mean absolute prediction error assessed for sigma values from 0.001 to 1. Lower plot shows the range of beta estimates associated with each sigma in different colors for each beta value. Sigma = 0.1 minimized the absolute prediction error of the beta estimates for the covariates and was used for the main model.

We calculated the mean absolute prediction error (MAPE) from an eightfold cross‐validation as a measure of the predictive ability of our model (Hooten & Hobbs, 2015). We divided the dataset into eight clusters. We then withheld one data cluster from each model run and compared the predicted values from the model to the recorded data values of the withheld data. We used normal priors centered on 0 for all of our beta coefficients. The prior for $\beta_{0}$ had a variance of 10. For all other beta coefficients, we used Bayesian regularization to optimize the variance on the priors to minimize the MAPE (Hooten & Hobbs, 2015). We performed an iterative search, fitting the model with variances of 0.001, 0.01, 0.05, 0.1, 0.15, 0.2, 0.5, and 1. This allowed for selection of the variance with the minimum MAPE across model runs (Appendix A; Figure A1). We then ran the model with the whole dataset, using the best optimized variance for the priors. For the shape parameter of the negative binomial, we used a uniform prior from 0 to 500. The model was run with a burn‐in of 5000 iterations followed by additional 10,000 iterations. We also calculated Moran's I (Gittleman & Kot, 1990) to test for spatial autocorrelation in the residuals.

### 2.6 fCort and demographic rates

We included additional data collected from radio‐marked birds to estimate the effects of fCort on adult survival, breeding propensity, nest success, and overall breeding success using a Bayesian framework. For adult female survival, we used a known fate binomial survival model with a weekly interval (Royle & Dorazio, 2008). We also included seasonal offsets, a 1 or 0 assigned for that occasion, on survival for the spring (March 1–May 31), summer (June 1–July 31), fall (August 1–October 31), and winter (November 1–February 28/29). We only modeled survival during the first year following collection of a feather sample. We modeled probability of survival for each individual female, i, at time, t, as:

$$\begin{array}{cc} \log\mathrm{it}\left(s_{i,t}\right)= & \beta_{0}+\beta_{\mathrm{cor}t}\times {\mathrm{cor}t}_{i}+\beta_{\mathrm{age}}\times {\mathrm{age}}_{i}\\ & +\beta_{\text{spring}}\times {\text{spring}}_{t}+\beta_{\text{summer}}\times {\text{summer}}_{t}+\beta_{\text{fall}}\times {\text{fall}}_{t}. \end{array}$$

The winter season served as the intercept. Birds were assigned a 1 for age if first sampled as adults, or a 0 if sampled as yearlings, thus $\beta_{\mathrm{age}}$ reflects the difference between adults and yearlings.

For each female, i, we investigated whether fCort affected different components of the recruitment process. Breeding propensity ($y_{\mathrm{bp},i}$) was recorded as a 1 if we observed a female on a nest, and a 0 if we never observed a nesting attempt. Similarly, nest success ($y_{\mathrm{ns},i}$) was recorded as a 1 if birds successfully hatched a nest, and a 0 otherwise. We modeled the probability of success for each component of the recruitment process (d) as $y_{d,i}\sim \text{Bernoulli}\left(p_{d,i}\right)$, where,

$$\log\mathrm{it}\left(p_{d,i}\right)=\beta_{d,0}+\beta_{d,\mathrm{cor}t}\times {\mathrm{cor}t}_{i}+\beta_{d,\mathrm{age}}\times {\mathrm{age}}_{i}$$

Overall breeding success ($y_{\mathrm{bs},i}$) was considered to be a 1 if the bird fledged, raised to 42 days, at least one chick. To estimate the number of chicks that each female successfully fledged $\left(y_{f,i}\right)$, we modeled $y_{f,i}$ ~ $\text{Zero}-\text{Inflated Poisson}\;\left({\mathrm{bs}}_{i}\times \mu \;\right)\;$where μ was the mean and variance of the number of chicks fledged, based on,

$$\log\left(y_{f,i}\right)=\beta_{f,0}+\beta_{f,\mathrm{cor}t}\times {\mathrm{cor}t}_{i}+\beta_{f,\mathrm{age}}\times {\mathrm{age}}_{i}$$

## 3 RESULTS

### 3.1 Corticosterone model

**FIGURE 3:**
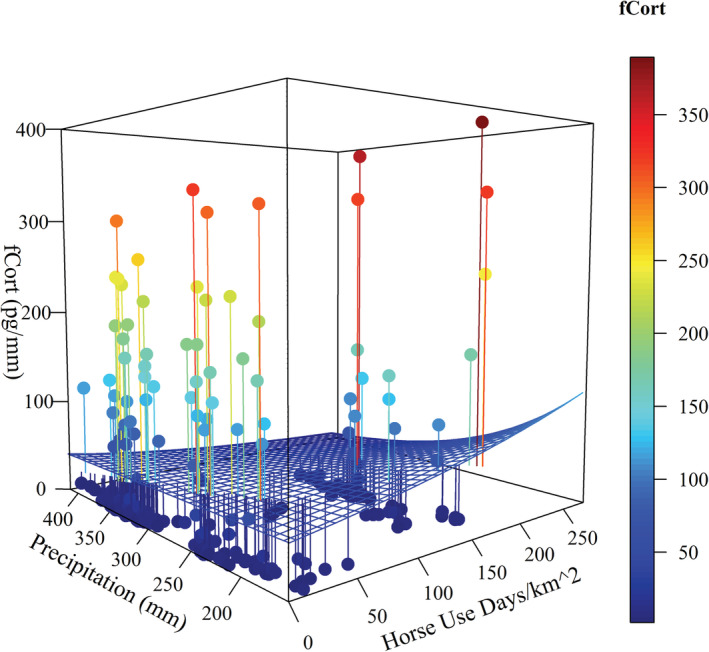
Interaction effects of horses (horse use days per km^2^) and annual precipitation (mm) on fCort (pg/mm feather), at the mean of all other covariates. Grid surface represents the estimated interaction of effects from the full model. Points are observed values of fCort with lines to corresponding location on the grid surface; right color bar represents these fCort measurements in pg/mm.

**FIGURE 4:**
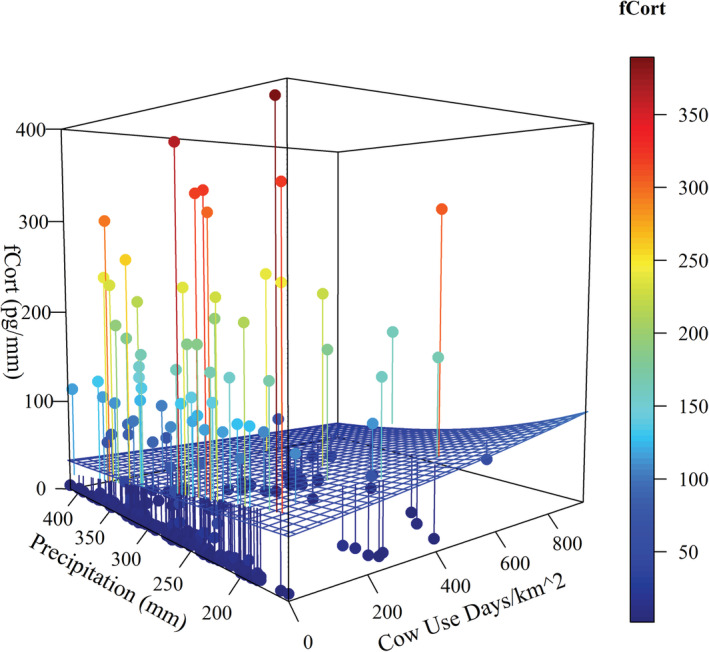
Interaction effects of cows (cow use days per km^2^) and annual precipitation (mm) on fCort (pg/mm feather), at the mean of all other covariates. Grid surface represents the estimated interaction of effects from the full model. Points are observed values of fCort with lines to corresponding location on the grid surface; right color bar represents these fCort measurements in pg/mm.

**FIGURE 5:**
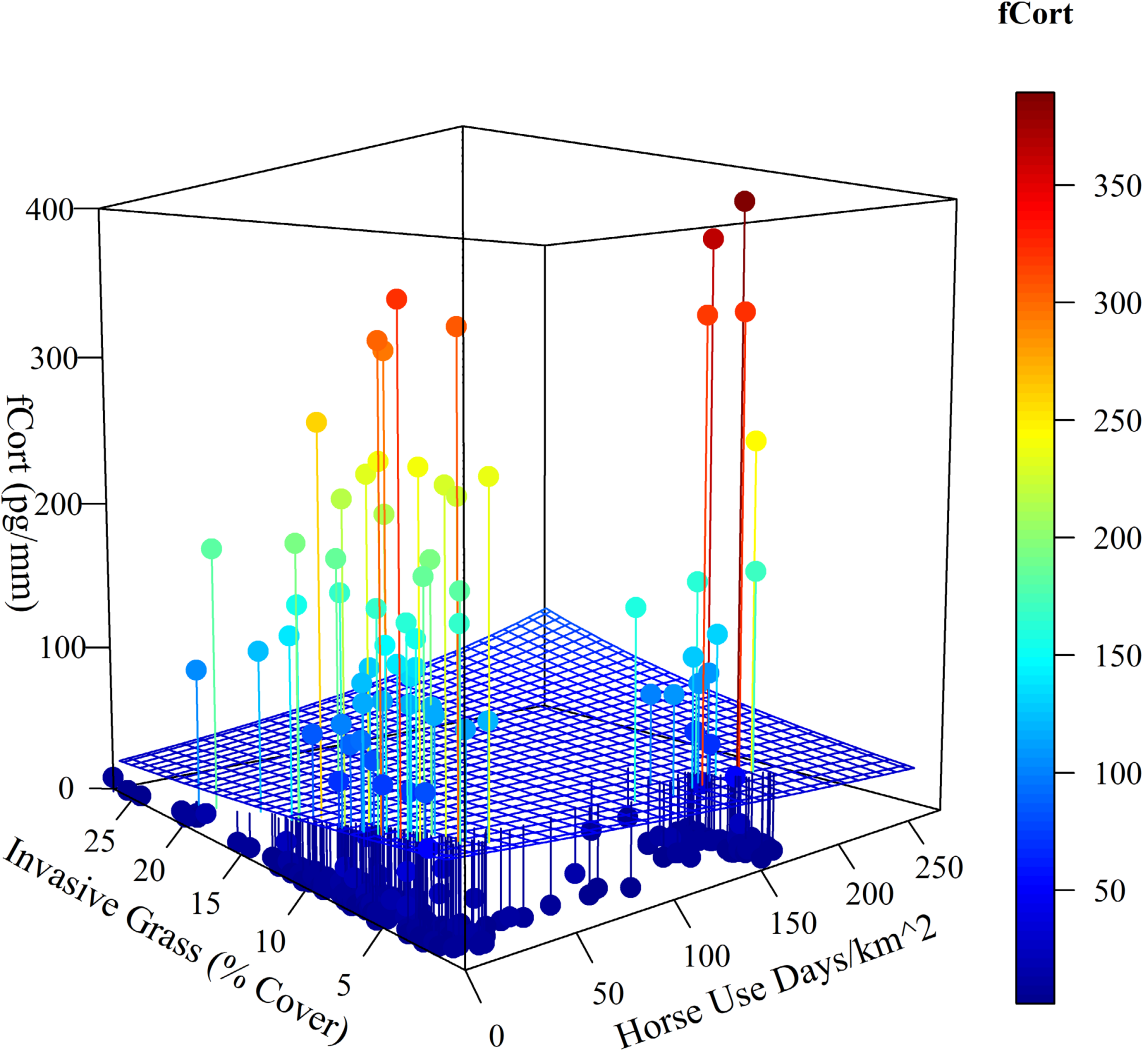
Interaction effects of horses (horse use days per km^2^) and annual grass cover (% cover) on fCort (pg/mm feather), at the mean of all other covariates. Grid surface represents the estimated interaction of effects from the full model. Points are observed values of fCort with lines to corresponding location on the grid surface; right color bar represents these fCort measurements in pg/mm.

We estimated fCort for 280 female sage‐grouse. We detected substantial among‐individual variation in fCort levels, with 65% of the sample having fCort levels <10 pg/mm, while 20% of the sample had fCort levels >100 pg/mm feather. There was no effect of age, and body condition only had a small negative effect on mean fCort (Table 1). In contrast, horse and cow densities interacted with precipitation to influence fCort levels. Increasing numbers of horses were associated with higher fCort in drought years (Figure 3; Table 1). This interaction was similarly positive, but the effect was not as large for cows (Figure 4; Table 1). FCort levels were also positively associated with the interaction between horse density and percent cover of annual grasses (Figure 5; Table 1). Despite substantial among‐individual variation, fCort levels tended to be highest in grazing allotments or horse management areas containing the largest numbers of cows and horses, respectively. We found no evidence of spatial autocorrelation in the variables, as indicated by a nonsignificant Moran's I (Moran's I −0.01, SD = 0.023, *p* = .759).

**TABLE 1:** Model estimates for the full fCort model with covariates

Term	Estimate	SD	LCI	UCI	Pr < 0	Pr > 0
Intercept	3.779	0.107	3.574	3.99	0	1
Cows	0.016	0.064	−0.107	0.143	0.405	0.595
Horses	−0.056	0.077	−0.206	0.096	0.764	0.236
Body condition	−0.037	0.061	−0.159	0.081	0.726	0.274
Age	−0.004	0.087	−0.174	0.168	0.517	0.483
Annual grass	−0.078	0.073	−0.219	0.065	0.86	0.14
Annual precipitation	−0.125	0.068	−0.258	0.008	0.967	0.033
Horses × precipitation	−0.125	0.069	−0.261	0.01	0.965	0.035
Horses × annual grass	0.075	0.086	−0.095	0.243	0.19	0.81
Cows × precipitation	−0.043	0.06	−0.161	0.073	0.759	0.241

*Note*: LCI and UCI represent lower and upper 95% credible intervals, respectively. Pr < 0 and Pr > 0 were the proportions of the posterior distribution below and above zero, respectively.

### 3.2 Demographic model

**FIGURE 6:**
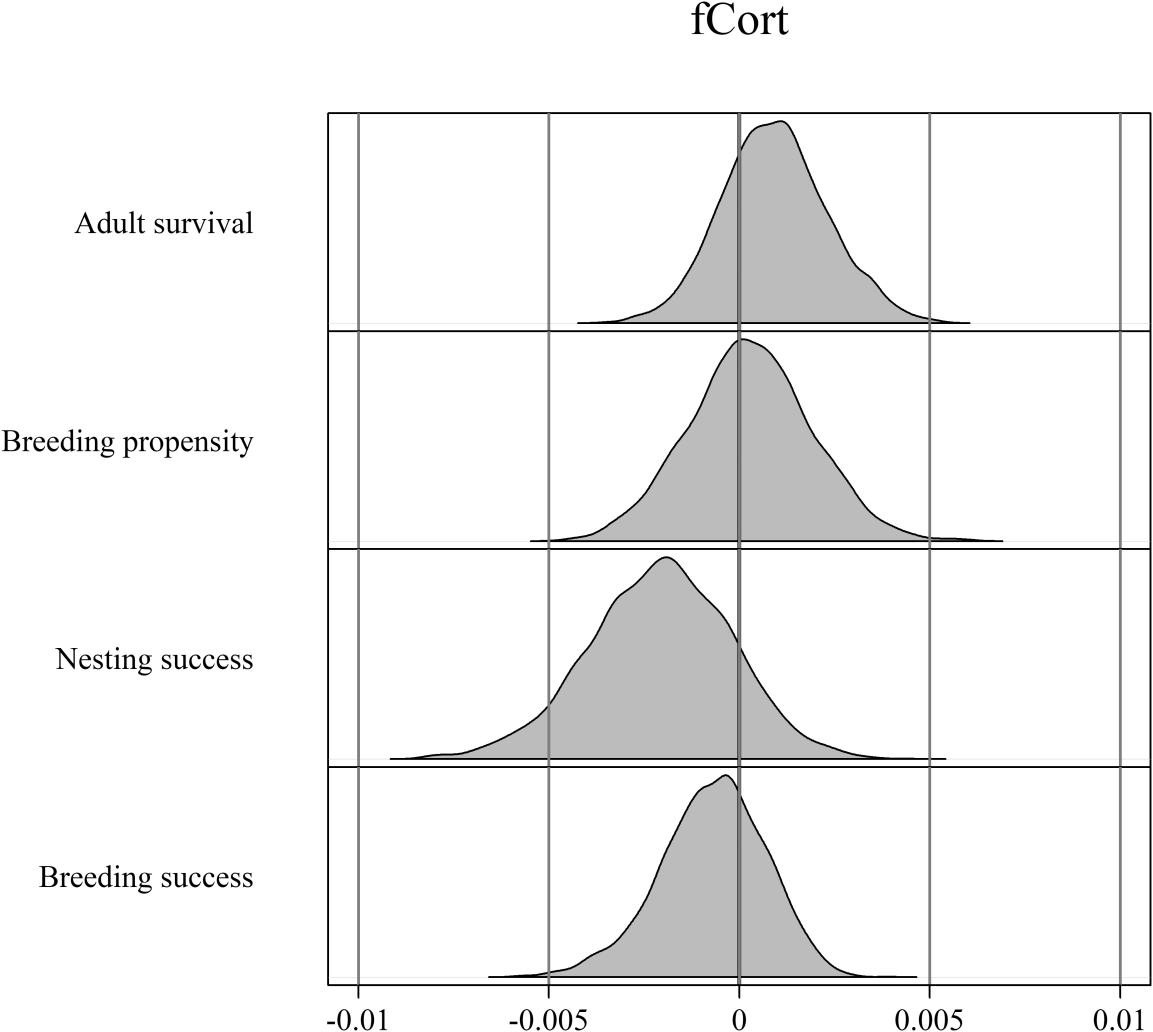
Posterior distributions for parameters from the models using fCort as a predictor of demographic rates—adult survival (known‐fate binomial), breeding propensity (Bernoulli), nest success (Bernoulli), and overall breeding success (zero‐inflated Poisson and Bernoulli) in the breeding year following fCort deposition (Table 2).

Mean fCort was not strongly predictive of future survival or reproductive success (Table 2). The posterior distribution for the beta estimate for nest success, however, suggested the potential for a negative relationship between fCort and nest success (Figure 6; Table 2).

**TABLE 2:** Estimated effects of fCort on each demographic rate

Demographic rate	Estimate	SD	LCI	UCI	Pr < 0	Pr > 0
Adult survival	0.001	0.001	−0.002	0.004	0.252	0.748
Breeding probability	0	0.002	−0.003	0.003	0.435	0.565
Nest success	−0.002	0.002	−0.006	0.001	0.873	0.127
Breeding success	−0.001	0.001	−0.004	0.002	0.691	0.309

*Note*: LCI and UCI represent lower and upper 95% credible intervals, respectively. Pr < 0 and Pr > 0 were the proportions of the posterior distribution below and above zero, respectively.

## 4 DISCUSSION

Mean fCort levels in female sage‐grouse were higher when density of non‐native ungulates was higher under drought conditions. Ungulates in arid environments can remove a high percentage of primary productivity (Baur et al., 2018; Veblen et al., 2015), an effect that is exacerbated under drought conditions when primary production is reduced (Donnelly et al., 2018; Zeigenfuss et al., 2014). Our findings are, therefore, consistent with the hypothesis that grazing by ungulates, likely through a reduction in late summer food availability for sage‐grouse (Street, 2020), acts as a chronic stressor for a significant number of female sage‐grouse. Horses in particular remove vegetation closer to the ground than other ungulates and consume upward of 20% more plant matter than cows (Hanley & Hanley, 1982). This may explain the stronger effect found with horses than with cows, in spite of the higher density of cows on the landscape. The increase in fCort correlated with the combined effect of horses and annual grass cover is not suprising, given that horses can help spread cheatgrass (King et al., 2019) and sage‐grouse recruitment suffers in areas with higher cheatgrass cover (Blomberg et al., 2012), as cheatgrass expansion displaces critical native perennial grasses and forbs (Williamson et al., 2020).

Our findings are consistent with other research demonstrating that environmental or social restrictions on energy or nutrient intake can produce chronic physiological stress (Fairhurst et al., 2017; Johns et al., 2018; Will et al., 2015; Wingfield et al., 1997; Wingfield et al., 2017; Wingfield & Sapolsky, 2003). Drought conditions are known to reduce reproductive investment by female sage‐grouse (Blomberg et al., 2012; Blomberg et al., 2017; Blomberg, Sedinger, et al., 2013a), and male sage‐grouse were less likely to attend leks during drought, especially at higher population densities (Blomberg, Sedinger, et al., 2013b), indicating that factors reducing food availability between breeding seasons constrain reproductive activities in sage‐grouse. The fact that grazing by ungulates under drought conditions was associated with increased fCort levels is consistent with the general linkage between nutrient intake and elevated GCs, and elevated GC levels and reduced breeding investment (Hansen et al., 2016; Harms et al., 2015; Williams et al., 2008). We caution that our results, while consistent with the hypothesis that habitat degradation and food restriction elevate GCs in some females, should not be used to establish a direct linkage between a chronic physiological stress and reproductive investment because we assessed the GC levels several months before breeding began. Delayed impacts on current breeding and survival (carryover effects) have been demonstrated for other variables in various avian species, such as previous breeding experience (Souchay et al., 2014; Warren et al., 2014) and population density (Blomberg et al., 2017). We investigated the combination of precipitation and grazing on breeding propensity in female sage‐grouse elsewhere (Behnke, 2021). Our results suggest that ungulate use interacts with precipitation to negatively affect probability of breeding by female sage‐grouse but does not influence survival.

Our data suggest that not all individuals responded the same way to grazing impacts (Figures 2 and 3). This could result from underlying intrinsic differences in reactivity to stressors (Cockrem, 2007, 2013) or other aspects of individual quality. Unknown circumstantial influences during the period of feather growth such as a predator attack, prior breeding effort, or disease status could also contribute to variation in response (Wingfield et al., 2017). Females that attended a brood during the summer delay molt of their primary feathers (Braun et al., 2020). If replacement of body feathers is also delayed, the timing of fCort deposition could differ by previous reproductive state. However, we found no effect of age on fCort, thus adults showed similar levels to yearlings who had no previous reproductive experience.

We found weak support that fCort was related to decreased nesting success, in line with the Cort‐Fitness Hypothesis, but fCort was not otherwise predictive of vital rates. The time interval between the elevated GCs and nesting, however, suggests that both reflect nutritional condition, rather than the GC elevation causing lower nest success in some females. Because we captured females in spring of the year following feather growth, our sample only contains those females who survived from fall to spring. If high fCort levels had more immediate survival consequences, we would have been unable to detect them in this analysis because mortality would have occurred before females entered our demographic samples. The associations we have detected are, thus, likely a conservative measure of these relationships.

Using Bayesian methods to address the uncertainty in our estimates, we were able to identify the variation in female responses to stressors. We likely would have missed this important pattern with traditional methods used for comparing hormone levels. We observed a significant amount of variation in fCort among females. We modeled variation in the mean of the negative binomial distribution and allowed the rate (variance term) to be constant. We also attempted to model variation in the rate term of the negative binomial and with a Poisson distribution, but observed poorer fit. Jankowski et al. (2014) standardized fecal corticosterone so that they could fit a linear model with a normal distribution. The highly skewed distribution of fCort levels we found could have influenced conclusions in studies that assumed a normal distribution for the residual variance. We emphasize that this variation is important to our understanding of the physiological response of chronic stressors. Our results suggest that there will likely be females with elevated fCort levels even when chronic stress is low, and there will likely be females with low fCort levels when chronic stress is high. Yet, the proportion of birds with high GC levels is higher when chronic stress is higher, and that adverse weather conditions combined with higher levels of either livestock of feral horses represent one source of chronic stress in our system.

Climate projections for the Great Basin indicate more frequent and longer‐lasting drought (Bradford et al., 2020; Snyder et al., 2019). Our results suggest that these conditions, along with unchecked increases in feral horse populations, will contribute to chronic stress in a substantial proportion of sage‐grouse. Birds may acclimate to grazing pressure during wet or average years, but such responses are insufficient under drought conditions when resources become scarce. While short‐term spikes in GCs facilitate survival and onset of breeding, chronic stress can lead to suppression of immune response, reproduction, and growth. Downregulation of the stress response system due to chronic stress can impede an individual's ability to respond to acute stressors when needed (Rich & Romero, 2005). Our results suggest that under increased drought, female sage‐grouse could experience an increased frequency of physiological stress under current grazing regimes.

Our study used a large dataset of feather corticosterone across a key sage‐grouse habitat region of over 1 million hectares. This allowed us to assess both individual variation and effects of a gradient of biotic and abiotic environmental variables. Declines of sage‐grouse populations have continued (Coates et al., 2021) despite intensive conservation efforts over the last few decades. Investigating the physiological links between the birds, their habitat, and the threats to both is important for informing future conservation actions.

## AUTHOR CONTRIBUTIONS

**Tessa Behnke:** Conceptualization (equal); data curation (equal); formal analysis (equal); methodology (equal); writing – original draft (lead); writing – review and editing (lead). **Phillip Street:** Conceptualization (equal); data curation (equal); formal analysis (equal); methodology (equal); writing – original draft (supporting); writing – review and editing (supporting). **Scott Davies:** Data curation (equal); methodology (equal); writing – original draft (supporting); writing – review and editing (supporting). **Jenny Ouyang:** Conceptualization (equal); formal analysis (equal); methodology (equal); writing – original draft (supporting); writing – review and editing (equal). **James S. Sedinger:** Conceptualization (equal); formal analysis (equal); funding acquisition (equal); methodology (equal); writing – original draft (supporting); writing – review and editing (equal).

## CONFLICT OF INTEREST

The authors declare no commercial or financial relationships generating a conflict of interest in this work.

## Data Availability

Data and R code are archived in Dryad (https://doi.org/10.5061/dryad.02v6wwq63).
